# The Update on Instruments Used for Evaluation of Comorbidities in Total Hip Arthroplasty

**DOI:** 10.1007/s43465-021-00357-x

**Published:** 2021-01-26

**Authors:** Łukasz Pulik, Michał Podgajny, Wiktor Kaczyński, Sylwia Sarzyńska, Paweł Łęgosz

**Affiliations:** 1 Department of Orthopedics and Traumatology, Medical University of Warsaw, Lindley 4 St, 02-005 Warsaw, Poland; 2 Student Scientific Association of Reconstructive and Oncology Orthopedics of the Department of Orthopedics and Traumatology, Medical University of Warsaw, Warsaw, Poland

**Keywords:** Arthroplasty, Replacement, Hip, Osteoarthritis, Orthopedics, Comorbidity, Multimorbidity, Chronic diseases

## Abstract

**Introduction:**

It is a well-established fact that concomitant diseases can affect the outcome of total hip arthroplasty (THA). Therefore, careful preoperative assessment of a patient's comorbidity burden is a necessity, and it should be a part of routine screening as THA is associated with a significant number of complications. To measure the multimorbidity, dedicated clinical tools are used.

**Methods:**

The article is a systematic review of instruments used to evaluate comorbidities in THA studies. To create a list of available instruments for assessing patient's comorbidities, the search of medical databases (PubMed, Web of Science, Embase) for indices with proven impact on revision risk, adverse events, mortality, or patient's physical functioning was performed by two independent researchers.

**Results:**

The initial search led to identifying 564 articles from which 26 were included in this review. The measurement tools used were: The Charlson Comorbidity Index (18/26), Society of Anesthesiology classification (10/26), Elixhauser Comorbidity Method (6/26), and modified Frailty Index (5/26). The following outcomes were measured: quality of life and physical function (8/26), complications (10/26), mortality (8/26), length of stay (6/26), readmission (5/26), reoperation (2/26), satisfaction (2/26), blood transfusion (2/26), surgery delay or cancelation (1/26), cost of care (1/26), risk of falls (1/26), and use of painkillers (1/26). Further research resulted in a comprehensive list of eleven indices suitable for use in THA outcomes studies.

**Conclusion:**

The comorbidity assessment tools used in THA studies present a high heterogeneity level, and there is no particular system that has been uniformly adopted. This review can serve as a help and an essential guide for researchers in the field.

## Introduction

Total hip arthroplasty (THA) is performed in 200 patients per 100,000 population in Organisation for Economic Cooperation and Development (OECD) countries yearly, which makes it one of the most common orthopedic surgeries [1]. The number of patients undergoing THA is continually increasing, and THA's efficiency is on the rise [2]. One of the causes of increasing effectiveness is a better assessment of a patient's health status to provide more personalized treatment based on their risk factors. According to research, 83.7% of patients undergoing hip surgery suffer from comorbidities [3]. Researchers indicate that concomitant diseases can affect the outcome of THA, including postoperative complications, risk of reoperation, cost of patient's treatment, future mobility of the patient, and outcomes represented by joint-specific measures including: Western and McMaster Universities Osteoarthritis Index (WOMAC), the Hip Disability and Osteoarthritis Outcome Score (HOOS), the Harris Hip Score (HHP), the Oxford Hip Score (OHS) and the Mayo Hip Score (MHS) [4]. Hence, the in-depth evaluation of comorbidities is vital for predicting THA outcomes [5]. The comorbidity index used for clinical practice should have simple computation, and data used for estimating should be easy to obtain. Most comorbidity indices are based on the International Statistical Classification of Diseases and Related Health Problems (ICD-10) coding, which provides better data assembling. ICD-10 codes are also collected in medical databases, which could be helpful for population-based or retrospective studies. Data for creating comorbidity indices could be obtained from a patient's exam, medical history, or prescription data, and the diseases used for estimating comorbidity indices should have a high prevalence and proven impact on THA outcome. There are also attempts to quantify comorbidities' influence by using weights assigned to each comorbidity to provide better risk assessment. Demographic factors such as age, body mass index (BMI) are often included in comorbidity indices [6].

### Methods

The systemic search of medical databases Embase, PubMed, and Web of Science was conducted by two independent researcher’s MP and WK. To find the most valuable and recent data, we estimated the following search criteria: articles must be written in English, published between 2016 and 2020, and contain the following keywords: "HIP," "ARTHROPLASTY", "REPLACEMENT" linked with the keyword "COMORBIDITY INDEX" using the operator "AND". Articles in which THA was performed for femoral neck or acetabular fracture were excluded from research using the operator "NOT" and phrase "FRACTURE" in search criteria. Animal studies were also excluded using the operator "NOT" and the phrase "ANIMAL" and "ANIMALS". From the obtained literature collection, initial titles and abstracts selection were performed. The second step was to screen full texts and exclude publications that do not measure comorbidities' impact on THA outcomes and review articles. The last step was to choose publications that discuss the impact of comorbidity in clinical practice, including predicting postoperative complications, adverse events, physical status, quality of life revision rate, length of hospitalization, risk of readmission, and mortality in different periods. Data from the last collection was extracted into Table 1 to present a comprehensive overview of the most recent assessment tools. A search of reference lists of identified articles was performed to identify other relevant studies. This additional search aimed to find other, less often used indices, which could be a valuable tool for patient's health assessment.

**Table 1:** Comorbidity measurement tools used in THA studies

	Title	Author	Year	Study type	THA group	Index	Outcome	Results
1	Associations between comorbidity and quality of life outcomes after total joint replacement [59]	Snell, DL	2020	Prospective	142	ASA	WHOQOLBref (World Health Organisation Quality of Life 8-item index)	Number of comorbid conditions was a stronger predictor of WHOQOL-Bref than ASA
2.	The association between comorbidity and the risks and early benefits of total hip arthroplasty for hip osteoarthritis [57]	Mannion, AF	2020	Retrospective	1584	ASA CCI	Complications and severity of complications OHS (The Oxford Hip Score) Satisfaction	Higher ASA correlated with higher complication rate and severity of complications Higher ASA was associated with a worse OHS ASA showed no association with the single-item outcome satisfaction measures CCI does not provide any additional predictive value
3.	Predictors of the use of analgesic drugs 1 year after joint replacement: a single-centre analysis of 13,000 hip and knee replacements [60]	Rajamaki, TJ	2020	Retrospective	6238	CCI	Use of opioids and other analgesics 1 year after surgery	Higher CCI is a risk factor of increase postoperative use of analgesics CCI of two or more was associated with a higher risk ratio for the use of any analgesic drugs
4.	Modified frailty index as a predictor of the long-term functional result in patients undergoing primary total hip arthroplasty [18]	Pulik, Ł	2020	Retrospective	365	mFI-5 mFI-11	WOMAC (The Western Ontario and McMaster Universities Osteoarthritis Index) HHS (Harris Hip Score) VAS (Visual Analogue Scale) HKASS LOS (length of stay) Complication	The mFI-5 and mFI-11 are predictors of WOMAC and LOS
5.	Risk stratification in primary total joint arthroplasty: the current state of knowledge [11]	Gronbeck, C	2019	Retrospective	5251	ASA CCI	Complication Readmission Reoperation Mortality	ASA was a risk factor for most assessed outcomes* ASA was a more reliable risk stratification than CCI*
6.	New 5-factor modified frailty index predicts morbidity and mortality in primary hip and knee arthroplasty [61]	Traven, SA	2019	Retrospective	140158	mFI-5	Complications Surgical site infection Readmission Mortality	The mFI-5 is a predictor of postoperative complications, surgical site infection, readmission, 30-day mortality, and Complications*
7.	Frailty predicts medical complications, length of stay, readmission, and mortality in revision hip and knee arthroplasty [62]	Traven, SA	2019	Retrospective	13948†	mFI-5	Complications Readmission Mortality LOS	The mFI-5 is a predictor of complications, prolonged LOS, readmission, and mortality
8.	Predicting costs exceeding bundled payment targets for total joint arthroplasty [63]	Ryan, SP	2019	Retrospective	861	ECM, (modified) ASA	Cost of care	ECM and ASA are predictors of cost-of-care*
9.	A weighted index of elixhauser comorbidities for predicting 90-day readmission after total joint arthroplasty [64]	Goltz, DE	2019	Retrospective	4535	ECM weighted ECM unweighted	90-day readmissions	ECM weighted was not inferior to unweighted in the prediction of 90-day readmissions*
10.	Validation of the Mayo Hip Score: construct validity, reliability, and responsiveness to change [65]	Singh, JA	2019	Prospective	5307	ASA CCI	MHS (Mayo Hip Score)	ASA and CCI are successful predictors of MHS
11.	Predicting hospital length of stay and short-term function after hip or knee arthroplasty: are both performance and comorbidity measures useful? [66]	Poitras, S	2018	Prospective	54	CCI CCI08 (version 2008) ASA	LOS WOMAC OARS (Older Americans Resources and Services ADL questionnaire) TUG (Timed-Up-and-Go)	ASA show the ability to predict prolonged LOS* ASA, CCI, and CCI08 cannot predict patients function after 2 and 6 weeks*
12.	Discriminative ability of elixhauser's comorbidity measure is superior to other comorbidity scores for inpatient adverse outcomes after total hip arthroplasty [19]	Ondeck, NT	2018	Retrospective	68680	mFI CCI ECM	Myocardial infarction, pneumonia, sepsis Bleeding, pulmonary embolism, death Mechanical complications, infection Extended LOS Discharge to facility	ECM outperformed CCI and mFI in the prediction of all measured adverse outcomes
13.	Predicting adverse outcomes after total hip arthroplasty: a comparison of demographics, the american society of anesthesiologists class, the modified Charlson Comorbidity Index, and the Modified Frailty Index [58]	Ondeck, NT	2018	Retrospective	67792	ASA mFI-11 mCCI (modified)	Severe adverse event Minor adverse event LOS Discharge to a higher-level care center	ASA significantly outperformed mCCI and mFI in all investigated outcomes
14.	Is gain in health-related quality of life after a total hip arthroplasty dependent on the comorbidity burden? [67]	Glassou, EN	2018	Retrospective	1582	CCI	EQ-5D (EuroQol-5D)	Correlation of CCI and EQ-5D in 3-months follow up
15.	Rate and risk factors for periprosthetic joint infection among 36,494 primary total hip arthroplasties [68]	Triantafylopoulos, GK	2018	Retrospective	36494	CCI	PJI (periprosthetic joint infection)	CCI associated with PJI
16.	Preoperative risk factors for postoperative falls in persons undergoing hip or knee arthroplasty: a longitudinal study of data from the osteoarthritis initiative [69]	Riddle, DL	2018	Retrospective	596	CCI	Post-hospitalization falls	CCI influence the risk of 2 or more postoperative falls
17.	Is decreasing mortality in total hip and knee arthroplasty patients dependent on patients' comorbidity? [70]	Glassou, EN	2017	Retrospective	99962	CCI	90-days mortality	High CCI increased the risk of 90-day mortality
18.	Current risk adjustment and comorbidity index underperformance in predicting post-acute utilization and hospital readmissions after joint replacements: implications for comprehensive care for joint replacement model [36]	Kumar, A	2017	Retrospective	183578	CCI ECM	30, 60, 90-day readmission	Comorbidity indices show a weak association with hospital readmissions
19.	Higher modified Charlson Index Scores are associated with increased incidence of complications, transfusion events, and length of stay following revision hip arthroplasty [71]	Lakomkin, N	2017	Retrospective	6121†	mCCI ASA	Mortality Major complications Minor complications Transfusion Prolonged LOS	ASA classification was a predictor of mortality, major complications, transfusions, prolonged LOS but was not an independent risk factor for minor complications Higher preoperative mCCI scores were significantly associated with mortality, major complications, minor complication, rates of transfusion, and prolonged LOS
20.	Impact of comorbidities on outcome after total hip arthroplasty [72]	Loth, FL	2017	Retrospective	251	CCI	FJS-12 (Forgotten Joint Score-12) OHS SF-12 (Short Form-12)	CCI had an impact on preoperative pain, function, and joint awareness Postoperative improvement did not differ significantly between patients with and without comorbidities
21.	Incidence and risk factors for blood transfusion in total joint arthroplasty: analysis of a statewide database [73]	Slover, J	2017	Retrospective	83372	CCI	Blood transfusion	Odds of transfusion increased with the increasing number of comorbidities in the CCI*
22.	Risk adjusted mortality after hip replacement surgery: a retrospective study [74]	Messina, G	2017	Retrospective	25850	ECM	In-hospital and 30-day mortality	ECM is a predictor of in-hospital and 30-day mortality
23.	Comorbidity does not predict long-term mortality after total hip arthroplasty [24]	Bulow, E	2017	Retrospective	120836	CCI ECM	All-cause mortality	Demographic factors were better in mortality prediction than CCI and ECM
24.	Influence of comorbid conditions and low back pain on patient-reported outcome following total hip arthroplasty [74]	Hamilton, D.F.	2017	Retrospective	251	CCI	FJS-12 OHS SF-12	No statistically significant association of CCI with postoperative improvement in joint-specific outcomes
25.	Incidence of and preoperative risk factors for surgical delay in primary total hip arthroplasty: analysis from the American College of Surgeons National Surgical Quality Improvement Program [75]	Phruetthiphat, OA	2016	Retrospective	7750	CCI ASA	Delay of surgery	ASA and CCI were associated with surgery delay.
26.	Discharge destination after total joint arthroplasty: an analysis of postdischarge outcomes, placement risk factors, and recent trends [35]	Keswani, A	2016	Retrospective	41597	ASA	Discharge destination Readmission Severe adverse events	ASA is a predictor of no-home discharge destination, severe adverse events, and readmission

*The results also applied to total knee arthroplasty

^†^Revision arthroplasty

## Results

**Fig. 1:**
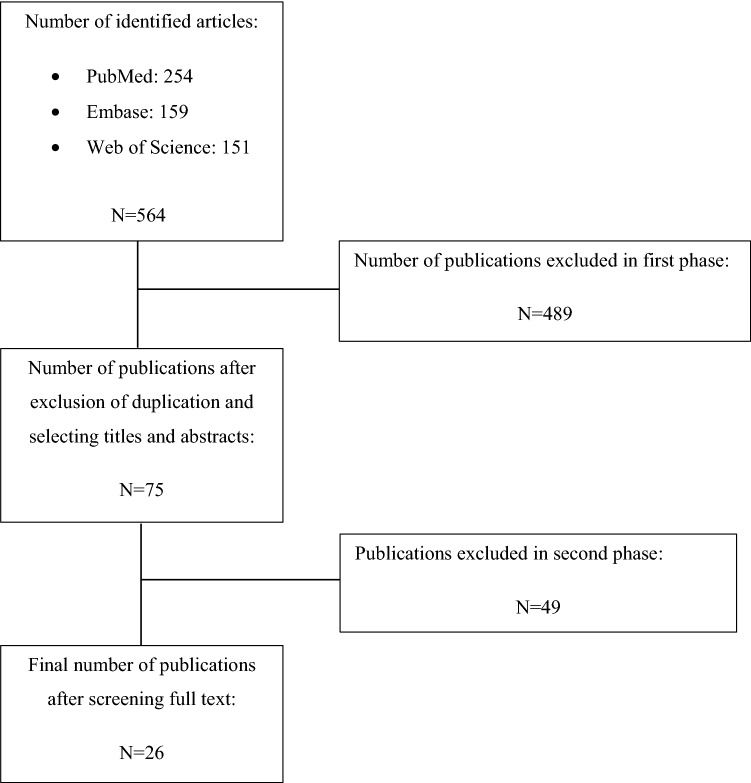
Summary of search and review process

The search resulted in the identification of 564 publications suitable for initial criteria. A further selection of the final 26 publications is presented in Fig. 1. In this review, the majority of publications (23/26) were retrospective studies. This systematic review's primary purpose was to find recently used tools for assessing a patient's comorbidity. The investigation revealed the following indices, presented with the frequency of their appearance: The Charlson Comorbidity Index (18/26), Society of Anesthesiology classification (10/26), Elixhauser Comorbidity Method (6/26), and modified Frailty Index (5/26). The following outcomes were measured: quality of life and physical function (8/26), complications (10/26), mortality (8/26), length of stay (6/26), readmission (5/26), reoperation (2/26), satisfaction (2/26), blood transfusion (2/26), surgery delay or cancelation (1/26), cost of care (1/26), risk of falls (1/26), and use of painkillers (1/26). The selected articles are listed in Table 1.

A Further examination of reference lists of 26 identified articles and combining them with systemic research resulted in creating a list of 11 indices suitable for predicting THA's outcome. The background information on the creation of each clinical tool and its essential characteristics is summarized in Table 2. The indices are subdivided into four categories depending on the tool’s scope. The index can be based on diagnosis, medical and demographic factors, prescription data, or general health status. The scoring method can vary between authors for the same clinical tool; in Table 3, the recommended scoring methods are described. Table 4 shows a detailed description of each instrument assessed in this review in the aspect of THA. The clinical conditions rated in each of the comorbidity indices are listed in Table 5. This systematic review revealed high heterogeneity in the methods used to assess THA patients' comorbidity, resulting from a lack of clinical guidelines.

**Table 2:** Background information on comorbidity measurement tools

Diagnosis-based	Charlson Comorbidity Index (CCI)
	The index allows the prognosis of the future health status the one-year mortality in patients suffering from multiple diseases. It was first introduced by Mary E. Charlson et al. in 1986 [7]. Although the CCI was initially developed to predict mortality after hospitalization, it has also been proven useful as a predictive tool for hospital readmission after orthopedic surgery [8]. This indicator may be valuable for physicians when treating a patient with multiple diseases [9]
	Modified Frailty Index (mFI)
	Frailty refers to patients declining physiological functioning related to age and comorbid diseases. Frailty presented as an index helps identify patients with an increased risk of postoperative complications. To evaluate the patient's frailty, The Canadian Study of Health and Aging Frailty Index (CSHA-FI) was created [14]. CSHA-FI consists of 70 variables. Each one represents the presence or absence of disease. It was simplified to Modified Frailty Index (mFI-11 and mFI-5) [15]
	Elixhauser Comorbidity Method (ECM)
	It consists of 30 variables, each representing a disorder based on a specific ICD code, and it can be easily obtained from medical records and datasets [19]. Conditions referred to in ECM have a high prevalence in patients undergoing THA [20]
	Cumulative Illness Rating Scale (CIRS)
	It was developed in 1968 by B. S. Linn [25]. It enables medical practitioners to assess the number and severity of comorbidities of their patients. CIRS ratings based on autopsy were highly predictive of analogous ratings based on historical data, proving the CIRS score's validity as an objective measure of physical illness burden [26]. CIRS was also suggested to be a better measure of multimorbidity than the Functional comorbidity index (FCI) and the CCI when the health-related quality of life (HRQOL) is the outcome of interest [27]
	Functional Comorbidity Index (FCI)
	The index is focused on predicting the patient's physical functioning as an outcome of a medical or surgical procedure [27]. It offers valuable information, especially for orthopedic research, including conditions like arthritis or osteoporosis. The functional status one year after the surgery could be measured based on Short Form 36 (SF-36) Patients Functioning Subscale (PFS) [29]
	The Index of Coexistent Disease (ICED)
	The ICED was developed by Greenfield et al. in 1993. It included the severity of functional impairment in addition to that of physical impairment. This method helps calculate the length of hospital stay and the risk of readmission [31]
Medical and demographic factors	Centers of Medicare and Medicaid developed Hierarchical Condition Category (CMS-HCC)
	Its purpose is to predict readmissions of operated patients to optimize the cost of treatment. It includes both demographic and clinical factors as concomitant diseases. Comorbidities included in CMS-HCC are based on ICD-9 coding [34]
	Readmission risk after a total hip replacement (RRATHR)
	The RRATHR was created to aggregate factors that could affect the risk of readmission after THA. RRATHR scale's purpose is to identify patients with a higher risk of complications to apply individualized care programs to improve readmission rate [37]
Prescription-based	The RxRisk-V score
	The RxRisk-V indicator measures comorbidity by using the patient's prescription data. Different approaches to evaluating multimorbidity using medication-based scores are being used to avoid adjusting data [39]. An index based on medication has some advantages over a diagnosis-based one. RxRisk is a medicine-based indicator, provides easier data assembling, is not affected by administrative misdiagnoses, and does not subject to variation of diagnosis coding systems. However, there is a risk of misclassification when the drug is used off-label. One medication included in the RxRisk measure could treat two simultaneous diseases leading to different scores in other scales [40]
General health status	The Charnley classification
	The Charnley classification was introduced in 1972 to assess an outcome of low-friction hip arthroplasties. Although the Charnley classification is not a proper comorbidity index, it is often used in the orthopedic literature. It is important to note that the Charnley classification considers the severity of comorbidities, making it unsuitable to use in studies based on medical records extraction [43]
	American Society of Anaesthesiology physical status classification (ASA)
	It is a widely used index for evaluating patients' physical status undergoing surgical procedures. The ASA provides reliable tools for assessing the patient's health status. Moreover, a higher ASA score correlates with prolonged surgery, longer hospitalization, increased readmission rate. It helps to optimize the cost of procedures by identifying patients who should receive more intensive perioperative care. Its strengths also include easy calculation, simplicity, clarity, and reference to the severity of the patient's condition, not only to the presence or absence of disease [48]. However, using the ASA score is criticized for the potential relativity of given scores because the ASA score is a subjective scale. Another weakness of the ASA score is that it describes only one aspect of a patient's condition and does not provide a comprehensive picture of its status [49]

**Table 3:** Scoring methods of comorbidity measurement tools

Diagnose-based	Charlson Comorbidity Index (CCI)
	Each of the 19 diseases is assigned a weight from 1 to 6. The index is the sum of the weights for each comorbid condition and can range from 0 to 33. There are many variations of the CCI, including the Charlson/Deyo, Charlson/Romano, Charlson/Halfon, and Charlson/Quan comorbidity indices. Each of them uses slightly different comorbidities. To calculate the 10-year survival rate, one needs to use the formula: 10-year survival = 0.983^ (eCCI × 0.9), where CCI = Charlson Comorbidity Index [10]
	Modified Frailty Index (mFI)
	The mFI consists of 11 or 5 factors; each one represents a health deficit. The total existing deficits are divided by the total number of all considered deficits. It was designed to obtain information on patient health status retrospectively from medical records and datasets [16]
	Elixhauser Comorbidity Method (ECM)
	The ECM should only be used as a combined score [19]. Van Walraven et al. propose a model in which ECM variables are used as a sum of weighted variables, but the created model does not outperform the unweighted score [21]. Kim et al. show that ECM performs better than other indices in predicting length of stay, mortality, complications, and discharge disposition [22]
	Cumulative Illness Rating Scale (CIRS)
	To calculate CIRS, one needs to rate each of 13 biological systems on a five-point severity scale. The score ranges from "0", meaning no impairment, to "4", for life-threatening impairment. The sum of ratings represents the evaluated comorbidity score [25]
	Functional Comorbidity Index (FCI)
	The patient is given one point for each of the 18 diseases associated with the declining patient's function, which are summed in a final score (0–18). FCI includes psychiatric impairments and obesity, which are not always included in more common comorbidity indices [27]
	The Index of Coexistent Disease (ICED)
	To assess the comorbidity with the Index of Coexistent Disease, one has to evaluate the patient's condition separately as per two different components [32]. The first one, Index of Disease Severity (IDS), comprises 17 categories of comorbid diseases, each of which is assessed on a 4-point scale, where "0" indicates the absence of disease and "3" indicates the disease's severe form. The other one, Index of Physical Impairment (IPI), measures the overall functional severity (disability) using a 3-point scale, where "0" means standard functionality, and "2" means the impossibility of functionality [33]. The composite Index of Coexistent Disease is then formed by collapsing various combinations of the two sub-indices into a 4-level classification [31]
Medical and demographic factors	Centers of Medicare and Medicaid developed Hierarchical Condition Category (CMS-HCC)
	It consists of 189 variables arranged in descending order of its severity. The number of variables is reduced to 70, excluding the least significant variables or variables with a smaller impact on the total cost. Variables are weighted and summed to create a total score [34]
	Readmission risk after a total hip replacement (RRATHR)
	The RRATHR scale consists of 16 variables combining two types of factors: demographic factors (age over 71 years, black race, first quartile income, Medicare or Medicaid payer status) and clinical factors (rheumatoid arthritis, obesity, hypertension, diabetes mellitus, chronic pulmonary disease, anemia, renal failure, fluid and electrolyte disorder, congestive heart failure, coagulopathy, and liver disease). To complete the score, factors are weighted. It is based on each factor associated with the readmission risk scale from 0 to 100 points [37]
Prescription-based	The RxRisk-V score
	The RxRisk-V consists of 46 variables, and each one represents the drug taken for a particular condition, and the weighting of RxRisk measures improves its predictive value [41]
General health status	The Charnley classification
	The Charnley classification divides patients into three classes by considering patient-specific factors [44]. Class A consists of patients with single joint arthropathy and no other comorbidity interfering with walking. Patients from class B suffer from bilateral arthropathy, but no other impairment or disease responsible for any defect in the ability to walk. Class C patients have multiple joint arthropathies or other locomotion factors, such as inflammatory arthritis, senility, hemiplegia, and cardiovascular or respiratory disability. In later studies, class B was suggested to be divided into B1, consisting of patients with their contralateral joint treated with arthroplasty, and B2, consisting of untreated patients [45]
	American Society of Anaesthesiology physical status classification (ASA)
	The ASA divides patients into six categories, but for THA evaluation, I–IV grades are used. Class I patients are healthy, class II have a mild systemic disease, class III have severe systemic disease. Class IV has a disease that poses a constant threat to life [50]. Patient condition is not described with ICD codes like in ECM, or CCI measures, which could lead to misclassification of patient diagnosis, difficulties in assembling necessary data for research purposes [51]

**Table 4:** The use of comorbidity measurement tools in total hip arthroplasty studies

Diagnosiss-based	Charlson Comorbidity Index (CCI)
	Comorbidity measures such as the CCI are appropriate to assess the prognosis in survival analyses. It is important to note that a summary measure may only be as good as the variables used to create it [11]. The most up-to-date and reliable version of the CCI used in surgical patients is the Royal College of Surgeons (RCS) modification [10]. However, there are more predictive indices for THA patients, such as the ASA Classification [12]. Similarly, the ECM was shown to be better at predicting inpatient death after orthopedic surgery. However, unlike other instruments, the CCI refers not only to the presence of comorbidity but also its severity. Therefore, it is the most frequently used comorbidity index in THA research [13]
	Modified Frailty Index (mFI)
	According to research, mFI appears to be a reliable index of predicting THA outcomes, including 30-day complications rate, reoperation risk, and length of stay and mortality [17]. The mFI-11 and mFI-5 can predict the long-term functional outcome of THA and hospitalization duration regardless of age [18]
	Elixhauser Comorbidity Method (ECM)
	Ondneck et al. study shows ECM's superiority over mFI and the CCI in predicting THA's adverse outcomes. The ECM outperformed demographic indicators, including age, which is the best demographic index of the procedure's outcome proven in medical practice in most groups presented in the study [23]. Another study by Mariano et al. proved that ECM outperforms CCI in predicting post-THA mortality, but the improvement was insignificant [13]. The ECM and other comorbidity measurement tools are a poor predictor of long-term THA mortality, and demographic indicators like age and sex outperform diagnose-based indicators in this study [24]
	Cumulative Illness Rating Scale (CIRS)
	The Cumulative Illness Rating Scale was found to be used as a comorbidity measure before total joint arthroplasty, including THA [28]
	Functional Comorbidity Index (FCI)
	Studies show that FCI is associated with a good predicting value compared to CCI when the outcome corresponds to the functional status [27]. FCI successfully predicts the patient's quality of life after THA [30]. Attempts at weighing the FCI assessment variables provide additional predictive value in patients with hip impairment [29]
	The Index of Coexistent Disease (ICED)
	Although the Index of Coexistent Disease is considered a valid and reliable method to measure comorbidity, it is not commonly found in the orthopedic literature. However, the ICED may prove useful for research purposes, as it was explicitly developed for orthopedic use [32]
Medical and demographic factors	Centers of Medicare and Medicaid developed Hierarchical Condition Category (CMS-HCC)
	Li et al. show that CMS-HCC without demographic factors has a higher predicting value of 6 months mortality than CCI and ECM [34]. A Higher CMS-HCC score is also associated with a higher cost of medical treatment [35]. However, Kumar et al. presented that CMS-HCC has the weak predictive ability of unplanned readmissions after 30, 60, 90 days in THA patients [36]
	Readmission risk after a total hip replacement (RRATHR)
	To our knowledge, there are no data proving its predictive value in THA outcomes. However, both demographic and clinical factors included in RRATHR have an impact on THA readmission risk [6, 38]
Prescription-based	The RxRisk-V score
	Inacio et al.'s studies show a high prevalence of conditions included in RxRisk-V score in patients undergoing THA, which is higher than the factors used in estimating ECM and CCI [42]. High-prevalent condition in THA patient is pain treated with anti-inflammatory medication (58.7% THAs), pain treated with opioids (55.0% THA), hypertension (56.0% THA), and anticoagulation disorders (53.0% THA) [44]. The medicine-based indicator provides good predictive value regards to mortality in patients undergoing THA. However, diagnosis-based one performs better in predicting 90-days and 1-year mortality [40]
General health status	The Charnley classification
	The Charnley classification can assess patients' preoperative health status undergoing THA [46]. It can influence the outcome of measures such as HHS, SF-36, the Nottingham Health Profile Score, and the. The Charnley class of patients may change over time due to the worsening of patients' pre-existent comorbidities or developing new ones [46]. It is also important to note that patient activity levels may be assessed using the Charnley classification [47]
	American Society of Anaesthesiology physical status classification (ASA)
	Schaeffer et al.'s study results indicate that patients with ASA score ≥ 3 have a 2.9 times higher risk of 30-day readmission after THA [49]. Almost half of the readmitted patients have an ASA score ≥ 3. Such patients are more prone to higher revision rates soon after THA (up to two years after the procedure) [49]. However, there is no connection between higher ASA scores and long-term revision rate [52]. The ASA score is also an indicator of complications, including endoprosthesis dislocation, pulmonary embolism, and more significant blood loss [53, 54]. Ridgeway et al. show an association between ASA score > 3 and 1.79 times higher risk of infection [55]. There is also a correlation between mortality after THA and ASA ≥ 3 [56]

**Table 5:** Clinical conditions rated in comorbidity indices

	Does the index rate include						
	Arterial hypertension	Diabetes mellitus	Rheumatoid arthritis	Neoplasm	Psychiatric disorders	Infectious diseases	Visual and hearing impairments
CCI [7]		✓	✓	✓			
ECM [19]	✓	✓	✓	✓	✓		
mFI11 [15]	✓	✓					
FCI [27]		✓	✓				✓
ICED [32]	✓	✓	✓	✓			✓
CIRS [25]	✓	✓	✓	✓	✓	✓	✓
RxRiskV [39]	✓	✓		✓	✓	✓	✓
RRATHR [37]	✓	✓	✓				
CMSHCC [34]		✓	✓	✓	✓	✓	

## Discussion

The THA is one of the most common surgeries worldwide that 1–3% of patients aged over 65 years will undergo in their lifetime [12]. Due to the high effectiveness in improving patients functioning and quality of life, the procedure was described in 2007 in "The Lancet" as "Operation of the Century" [80]. Currently, the age of patients undergoing THA increases, as is the comorbidity burden [81]. In a systematic review conducted by Buirs et al. [82], 11 out of 13 studies (84.62%) showed a significant negative relationship between comorbidities and postoperative hip function. In another review by Olthof et al. [83], multimorbidity predisposed to the longer hospital stay after THA, and in 8 out of 9 studies, the relationship was statistically significant. In all out of two eligible studies, comorbidities were associated with a higher cost of care. Also, cognitive status and mental health before surgery can affect the functioning after THA. Psychiatric disorders are associated with less satisfactory functional outcomes and less improvement in life quality, pain and satisfaction after surgery, prolonged hospitalization, complications, and increased mortality [84, 85]. Undeniably, the coexisting diseases can impact THA results, both traditional outcomes like mortality, risk of adverse events, or revision, and patient-oriented outcomes such as quality of life, physical function, and satisfaction [4]. Identifying patients at high risk of complications can lead to adequate qualification for the procedure and initiation of more rigorous prophylaxis. On the other hand, low-risk patients could be subjected to fast-track surgery, reducing the length of stay and care-related costs [58]. The current methods used to assess health status among patients qualified for THA are very diverse among the authors, making it difficult to compare individual results in a pooled analysis. This review is intended to facilitate the selection of the appropriate tool and its proper application. Table 6 represents the summary of the strengths and limitations of included comorbidity assessment methods.

**Table 6:** Strengths and weaknesses of comorbidity indices used in THA studies

Index	Strengths	Weaknesses
CCI [10, 13, 23]	Simple and good for international use Refers to severity of comorbidity	Worse for predicting perioperative adverse outcomes than ASA Worse at predicting inpatient death after orthopedic surgery than ECM
ASA [48, 49]	Refers to the severity of patient’s condition Popular, simple and easy to calculate	Subjective nature of the scale Does not provide a comprehensive picture of patient’s status Does not cover case complexity, mental health and physical functioning
ECM [13, 19, 23]	Best demographic index of the procedure’s outcome Better for predicting adverse outcomes in THA than mFI and CCI Better for predicting inpatient death after orthopedic surgery than CCI	Can cause difficulties in collecting and analysing data due to its complexity
mFI [15, 61]	Good for orthopedic surgery Can be predictive of the outcome of THA while containing just five factors	Does not relate to physical functioning
CIRS [26, 27]	Better measure of multimorbidity than the FCI and the CCI with HRQOL as the outcome of interest	Does not psychiatric disturbances highly prevalent in the elderly
FCI [27, 30]	Good predicting value corresponding to the functional status Predicts patient’s quality of life after THA	Worse for predicting mortality than CCI Doesn’t include the severity of comorbidity or rare disorders
ICED [32]	Explicitly developed for orthopedic use	Not commonly used in the orthopedic literature
CMS-HCC [22, 35, 36]	Can be used to estimate the cost of treatment Higher predicting value of 6 months mortality than CCI and ECM	Weak predictive ability of unplanned readmissions after 30, 60, 90 days The use of multiple variables could provide issues in index calculations and data collection
RRATHR [6]	Included factors have proven impact on readmission risk	No predictive value in THA
RxRisk-V [40]	Easy to assemble data Not affected by administrative diagnoses Is not affected by the differences in diagnosing coding systems	Being a medication-based index, it can lead to misclassifications
Charnley [47]	May be used to assess levels of patient activity	Does not take severity of comorbidities into consideration Not suitable for use in studies based on chart reviews or extraction of medical records

The most commonly used comorbidity measure in THA patients is the ASA classification, and the second one is the CCI. These clinical tools often serve as a reference point for measuring other indices' performance, including mFI and ECM. Both ASA and CCI can successfully predict the THA outcomes such as quality of life, physical function, complications, mortality, length of stay, and hospital readmission. Nevertheless, the ASA classification was more predictive than CCI when indices were compared in terms of adverse events (any, minor and serious), length of stay, and discharge to the higher level facility after THA. The ASA could better reflect patients' health status because of its dynamic assessment of comorbidities, while indices like CCI only note the presence of the disease. The CCI, an objective, diagnose-based measure, has less predictive power than a subjective tool like ASA. However, the ASA class had less discriminative ability than age in all the aforementioned outcomes. The available variants of CCI are presented in Table 7 [12, 57].

**Table 7:** Development and changes in CCI modifications

CCI modification	Development and changes
Deyo [76]	ICD-9-CM codes were assigned for each condition in the original CCI. The number of categories was reduced from 19 to 17
Halfon [77]	ICD-9-CM codes from the Deyo adaptation of the CCI were translated into ICD-10-codes
Romano [78]	ICD-9-CM codes were replaced with a set of codes, referred to as the Dartmouth–Manitoba codes, developed for use with the CCI
Schneeweiss [79]	Adjusted weights for the CCI conditions were introduced

The recent publications demonstrate that the ASA score has a good predictive value, but it could present significant discrepancies over time because of its dynamic and subjective nature [12]. That is why other indices like ECM are still under investigation. The ECM is based on ICD codes, which can be acquired from administrative data, unlike the ASA score, collected and assessed prospectively. The ECM is the third most commonly used comorbidity index in THA studies. It outperformed CCI and mFI to predict serious complications, e.g., sepsis, myocardial infarction, bleeding, mortality, mechanical complications, infection, extended length of stay, and discharge to the facility [28]. Also, comparing to ASA, it can be a better predictor of outcome after orthopedic surgery [86]. However, the complexity of 30 variables that could provide a broad perspective of the patient’s preoperative health status could lead to data collection difficulties. Using scores consisting of many variables could provide a situation when comorbidities with different impacts on THA are put on equal. That is why creating appropriate weights was made, but studies do not prove the additional utility of weighted scores [28].

Another example of an index that should also be considered in THA patients is the modified Frailty Index (mFI). With aging, the comorbidities burden increases, catabolic processes exacerbate, and the physiological reserve and resistance to stressors such as surgery declines. This state of organism exhaustion is referred to as frailty. The mFI is used to assess multimorbidity and frailty, and it is available in a version containing eleven components (mFI-11) and in a shortened version consisting of five items ("mFI-5"). Both versions effectively predict increased risk of prolonged hospitalization, complications, and reoperation after THA [61]. Due to its easy estimation, objectivity, and good predictive value of surgery outcomes, mFI is a promising clinical practice tool. It can be obtained retrospectively from medical records ICD coding. Previous studies have shown that mFI is a stronger predictor than age or ASA in predicting the length of hospitalization, complications, reoperation, and mortality after THA [17]. The mFI was recently proven to predict long-term functional outcomes (WOMAC) and length of hospital stay in patients after THA [18].

Other, less frequently used indices deliver a more diverse image of a patient's health status and provide additional predictive value than the beforementioned clinical tools. For example, the Functional Comorbidity Index (FCI) can predict postoperative patients' physical function and quality of life after THA. It includes aspects like obesity or mental status and focuses on physical function limitation. However, its predictive ability does not find reflection in recent studies, and it is not widely used in clinical practice. Moreover, The FCI, compared to CCI, has a worse predicting ability of mortality after THA [27]. Another less-commonly used index is RxRisk-V, a proven predictor of THA outcome with a unique calculation method based on a patient’s prescription data. The RxRisk-V provides good predictive value, as well as easy data collection. However, a medication-based index can lead to misclassifications when one medication is given to cure two comorbid diseases or medicament is given “off label” [42]. The Index of Coexistent Disease (ICED) is an example of an index considering both physical and functional status, but it is rarely used in orthopedic literature [32]. The Cumulative Illness Rating Scale (CIRS) differs from other indices because it rates each separate human body system. It could be a reliable and valid instrument for assessing comorbidity in THA patients. As a fast, objective, and easily quantified index, it is well suited to various research uses. [25]. As well as some lesser-known indices we presented in this review, demographic factors have a significant ability to predict the outcome of THA. Measurement tools like RRATHR and CMS-HCC combine demographic factors like age with comorbidities to create a more comprehensive reflection of a patient's health status. However, RRATHR was found to have no proven predictive value in THA, according to recent literature. Furthermore, their overwhelming complexity excludes them from everyday clinical practice instruments and adjusting care for patients' needs [54].

Studies discussing comorbidity indices' effectiveness highlighted that indices used in everyday practice should remain as easy as possible. Too many factors included in the index could lead to errors and hinder data assembling. Additionally, the index should be legible and straightforward for clinicians to provide a convenient and fast evaluation. That is why ASA and CCI are still widely used even though they do not precisely reflect a patient's health status. In opposition to more specific ones, general indices help assess which patient should receive more intensive peri/postoperative care. Using general indices also avoids the risk of equalizing different conditions in patients with the same comorbid disease [32]. Despite the variety of comorbidity assessment methods and measured outcomes, the majority of recent studies presented in this systemic review confirm the predicting ability of different comorbidity indices and convince that assessing patients' comorbid diseases is vital in clinical practice. This study does not contain all available comorbidity indices like Chronic disease score (CDS), Kaplan Feinstein Classification (KFC), Health-related Quality of Life Comorbidity Index (HRQL-CI) due to their absence in the orthopedic literature [87, 88].

## Conclusions

1. The most commonly used comorbidity indices in THA studies are CCI and ASA.
2. Currently, researchers focus not only on mortality and complications but also on the quality of life, function, and patient satisfaction after THA.
3. There is high heterogeneity in the methods used to assess the health status of THA patients.
4. Comorbidity indices should be an integral part of clinical practice because it allows predicting the risk of complications and the THA's functional outcome.
5. Less common comorbidity indices may also prove useful for researchers in THA studies.
